# Hypertension and Obesity as Cardiovascular Risk Factors among HIV Seropositive Patients in Western Kenya

**DOI:** 10.1371/journal.pone.0022288

**Published:** 2011-07-14

**Authors:** Gerald S. Bloomfield, Joseph W. Hogan, Alfred Keter, Edwin Sang, E. Jane Carter, Eric J. Velazquez, Sylvester Kimaiyo

**Affiliations:** 1 Division of Cardiology and Duke Clinical Research Institute, Duke University, Durham, North Carolina, United States of America; 2 Department of Biostatistics, Brown University, Providence, Rhode Island, United States of America; 3 Department of Medicine, Moi University School of Medicine, Eldoret, Kenya; 4 Divisions of Infectious Diseases and Pulmonary Medicine, Alpert School of Medicine at Brown University, Providence, Rhode Island, United States of America; Lerner Research Institute, Cleveland Clinic, United States of America

## Abstract

**Background:**

There is increased risk of cardiovascular disease among HIV seropositive individuals. The prevalence of HIV is highest in sub-Saharan Africa; however, HIV-related cardiovascular risk research is largely derived from developed country settings. Herein, we describe the prevalence of hypertension and obesity in a large HIV treatment program in Kenya.

**Methods:**

We performed a retrospective analysis of the electronic medical records of a large HIV treatment program in Western Kenya between 2006 and 2009. We calculated the prevalence of hypertension and obesity among HIV+ adults as well as utilized multiple logistic regression analyses to examine the relationship between clinical characteristics, HIV-related characteristics, and hypertension.

**Results:**

Our final sample size was 12,194. The median systolic/diastolic blood pressures were similar for both sexes (male: 110/70 mmHg, female: 110/70 mmHg). The prevalence of hypertension among men and women were 11.2% and 7.4%, respectively. Eleven percent of men and 22.6% of women were overweight/obese (body mass index ≥25 kg/m^2^). Ordinal logistic regression analyses showed that overweight/obesity was more strongly associated with hypertension among HIV+ men (OR 2.41, 95% CI 1.88–3.09) than a higher successive age category (OR 1.62, 95% CI 1.40–1.87 comparing 16–35, 36–45 and >45 years categories). Among women, higher age category and overweight/obesity were most strongly associated with hypertension (age category: OR 2.21, 95% CI 1.95–2.50, overweight/obesity: OR 1.80, 95% CI 1.50–2.16). Length of time on protease inhibitors was not found to be related to hypertension for men (OR 1.62, 95% CI 0.42–6.20) or women (OR 1.17, 95% CI 0.37–2.65) after adjustment for CD4 count, age and BMI.

**Conclusion:**

In Western Kenya, there is a high prevalence of hypertension and overweight/obesity among HIV+ patients with differences observed between men and women. The care of HIV+ patients in sub-Saharan Africa should also include both identification and management of associated cardiovascular risk factors.

## Introduction

The currently available evidence suggests that there is an excess risk of cardiovascular disease (CVD) among human immunodeficiency virus (HIV) seropositive (+) compared to HIV seronegative (−) individuals [1]. HIV+ patients are at an elevated risk of developing dyslipidemia, carotid atherosclerosis, large vessel vascular disease, coronary calcification, and coronary artery disease with or without anti-retroviral therapy [2], [3], [4], [5]. It has been suggested that of the many factors that are involved in the development of CVD in HIV+ patients, traditional cardiovascular risk factors are the main contributors [6]. The few studies evaluating the prevalence of traditional cardiovascular risk factors among HIV+ patients in sub-Saharan Africa suggest that rates are significantly lower than in developed countries but some of this difference may be attributed to underdetection based on low clinical suspicion and small sample sizes [7], [8], [9]. Large international studies have also implicated the HIV itself and anti-retroviral therapy (ART) as potential mediators of this increased risk of CVD [10], [11], [12], [13]. These studies, however, have not included patients from sub-Saharan Africa.

The impact of the HIV/AIDS epidemic has been the greatest in sub-Saharan Africa with 67% (22.1 million) of all people infected with HIV worldwide residing in sub-Saharan Africa and the majority of deaths globally due to HIV occurring in this region [14]. Concurrently, the epidemiology and demography of sub-Saharan Africa is shifting towards older populations with a higher proportion of CVD due to chronic, non-communicable diseases [15]. Despite these facts, the region is under-represented in studies examining the relationship between HIV and CVD risk factors. Understanding the distribution of cardiovascular risk factors among HIV+ patients in sub-Saharan Africa is important due to the known cardiovascular effects of HIV and its treatment, and the exceedingly high prevalence of HIV in this region. Furthermore, global efforts in sub-Saharan Africa aimed solely at HIV care may be missing a critical opportunity to improve overall cardiovascular health if chronic CVD risk factors are highly prevalent. The objective of this study was to describe the prevalence of hypertension, overweight/obesity and HIV-related cardiovascular risk factors in a large HIV treatment program in Western Kenya.

## Methods

### Ethics Statement

This retrospective study used de-identified data from the electronic medical records of HIV+ adult patients treated in the Academic Model Providing Access to Healthcare (AMPATH) program. Individual informed consent was not obtained. The Institutional Research and Ethics Committee of the Moi University School of Medicine and the Institutional Review Boards of Indiana, Duke and Brown Universities approved use of these data and waiver of informed consent.

### Study Site & Population

The AMPATH (previously an acronym for Academic Model for the Prevention and Treatment of HIV/AIDS) clinical care system was created in 2001 and has been described previously [16], [17]. AMPATH now provides HIV care and treatment to over 50,000 adults and children living with HIV/AIDS in 19 clinics throughout western Kenya. Patients are managed according to National Kenyan protocols, which are consistent with World Health Organization guidelines. Clinic visits occur monthly for all patients on ART unless alternative arrangements have been made with their health care provider. Patients who are not yet eligible for treatment are seen monthly or bi-monthly depending on their immunologic status and other factors in their health profile. Standard paper data collection forms are used at enrollment to the program and at each subsequent visit. Data from these forms are entered into the AMPATH electronic Medical Record System by data entry technicians [18]. Blood pressure was measured during a clinical encounter and was commonly performed using a manual sphygmomanometer by a trained nurse. Height and weight were also measured as part of routine HIV care. The anthropometric measurements were not measured according to a standard protocol but were performed as a part of routine clinical care.

**Figure 1:**
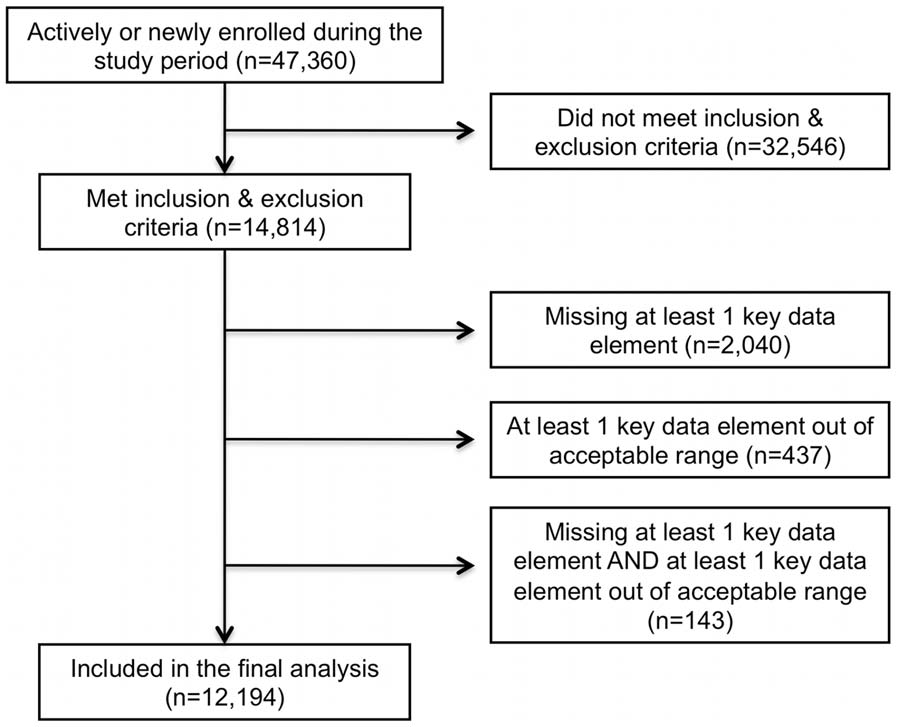
Analysis profile. The records of all patients enrolled in the AMPATH program between September 1, 2006 and March 5, 2009 were screened for inclusion and exclusion criteria described in the text. We also excluded patients who were missing data for any of the following elements: CD4 count, heart rate, height or weight. We also excluded patients who had vital data, which was thought to be out of acceptable range. Acceptable ranges were defined as: SBP>65 and <220 mmHg, DBP>40 and <120 mmHg, BMI>15 and <40 kg/m2 and heart rate >40 and <230 beats per minute. Some records could have had missing values for one variable and out of range for another. A total of 2,620 records were excluded for out of range or missing data yielding 12,194 records in the final analysis.

We included in this analysis HIV+ patients ages 16 to 80 years who were enrolled in the AMPATH program between September 1, 2006 and March 5, 2009. We excluded patients for whom only one or no blood pressure readings were recorded during the study period or who were pregnant during this time period. We further restricted our sample to those patients with available data height, weight, heart rate and CD4 count during the follow-up period. Clinical and laboratory data for each patient (blood pressure, weight, height, heart rate, creatinine) were drawn from the most recently available patient encounter for each variable separately. We included CD4 counts within 6 months of the last follow-up date in the analyses. There were 47,360 patients who were enrolled in the AMPATH program during the study period. Of these, 32,546 were excluded for the following reasons: 8,661 women were pregnant and 23,885 had insufficient blood pressure recordings during the study period. After applying inclusion and exclusion criteria, there were 14,814 patients. There were 437 patients (126 male, 311 female) who had data that were considered out of acceptable range for at least one of the key data elements and therefore not included, using the following criteria: systolic blood pressure (SBP)<65 or >220 mmHg, diastolic blood pressure (DBP)<40 or >120 mmHg, body mass index (BMI)<15 or >40 kg/m^2^, heart rate <40 or >230 beats per minute. There were 2,040 patients (707 male, 1,333 female) who were missing data for CD4 count, pulse or BMI and were excluded from the analysis. There were 143 patients who had values that were out of range for one of the key data elements as well as data missing for another key data element. A total of 2,620 patients were excluded due to missing data or data that was out of range. After applying inclusion and exclusion criteria, our final analysis consisted of 12,194 individuals. (Figure 1)

### Variable Definitions and Statistical Analysis

Prehypertension was defined as SBP 120–139 mmHg or DBP 80–89 mmHg. Hypertension was defined as SBP≥140 mmHg or DBP≥90 mmHg as of the most recent patient encounter. Use of protease inhibitors (PIs) was defined as being prescribed any of the locally available PIs which include lopinavir, ritonavir or darunavir. BMI was defined and categorized according to standard international definitions [19]. Creatinine measurements in micromoles per liter were converted to milligrams per deciliter by dividing by 88.4. Glomerular filtration rate was estimated using the abbreviated Modified Diet in Renal Disease equation [20]. We created a variable that represented the sum total cardiovascular risk that assigns one point for each of the following risk factors: SBP≥140 mmHg, DBP≥90 mmHg and BMI≥25 kg/m^2^, yielding a maximum value of 3. For the analyses including use of PIs, the sample sizes used were smaller due to a smaller number of patients for whom these data were available. Descriptive analyses were performed to display the overall distribution of the cardiovascular, clinical and HIV-related risk factors for men and women. Continuous variables are displayed as median (interquartile range) for our descriptive analyses and categorical variables are expressed as proportions.

Our analyses are designed to meet two goals: first, to describe the distribution of individual CVD risk factors over a joint categorization of age and CD4 count (using categories defined above); and second, to model the variation in CVD risk as a function of age, CD4 count, BMI, and duration of PI usage. Our analyses are stratified by gender.

To meet the first objective, we generated tables of mean SBP, DBP, BMI, and days on PI, and prevalence of hypertension, jointly stratified by CD4 category (CD4<200, 200≤CD4≤350, CD4>350) and age category (16≤age≤35, 35<age≤45, age>45 years). These age categories were based on the age distribution and life expectancy in Kenya (54 years in 2007) [21] and then selected by extrapolation to select the middle and older age cut-offs. We used the Kruskal-Wallis test to compare the distributions of SBP, DBP, BMI and duration of PI use across CD4 count categories within each age category. The Chi Square test was used to test for homogeneity of proportions of hypertensive subjects across CD4 categories within each age category. The Cochran-Armitage test was used to test for trend across SBP, DBP, BMI, duration of PI use and proportion of hypertensive subjects across CD4 count categories within each age category.

For a more concise summary of these associations, and to test hypotheses about factors associated with CVD risk, we used regression-based analyses. First, we used ordinal logistic regression models to model cardiovascular risk (range 0–3) as a function of CD4 count and age. Secondly, we used standard logistic regression to model the individual risk factors DBP≥90 mmHg, SBP≥140 mmHg, and HTN as a function of age, CD4 count and BMI. These models were fit separately for men and women. In the regression models, age was analyzed as three ordinal categories such that the odds ratios reflect the change in odds for one successively higher age category. CD4 count was categorized as: ≥200 or <200 cells/mm^3^, and BMI as ≥25 or <25 kg/m^2^. For the subset of patients prescribed PIs, we fit the same regressions described above but added use of PIs≥540 days as a covariate [12], [22].

Using thresholds for SBP (≥140 mmHg), DBP (≥90 mmHg), BMI (≥25 kg/m^2^), and length of time using PIs (≥540 days) we calculated the proportions of individuals exceeding the thresholds.

## Results

Baseline descriptive characteristics are shown in Table 1. The median (IQR) age was 43 (37–49) and 40 (34–47) years for men and women, respectively. Median SBP, DBP and BMI were similar between men (110 mmHg, 70 mmHg and 20.8 kg/m^2^) and women (110 mmHg, 70 mmHg and 22.0 Kg/m^2^), respectively. Prehypertension was present in 765 (17.8%) men and 1,075 (13.6%) women. Hypertension was present in 481 (11.2%) men and 583 (7.4%) women. Using a BMI threshold of ≥25 kg/m^2^, 453 (10.6%) men and 1,782 (22.6%) women were overweight/obese. A small number of men (226) and women (295) were prescribed a PI drug during this time period. Among patients taking PIs, the prevalences of overweight/obesity among men and women were 14.6% and 22.0%, respectively. In the subgroup of patients taking PIs, hypertension was present in 10.2% of men and 8.8% of women. Among patients who had been prescribed PIs, 54.4% of men and 54.6% of women had been taking a drug in this class for greater than 540 days.

**Table 1:** Summary of Clinical Characteristics.

	Men	Women
	n = 4,293 (35.2%)	n = 7,901 (64.8%)
	Median (IQR) or n (%)	Median (IQR) or n (%)
**Demographic and Clinical Characteristics**		
Age (years)	43	40
	(37–49)	(34–47)
Systolic BP (mm Hg)	110	110
	(100–120)	(100–120)
Diastolic BP (mm Hg)	70	70
	(60–80)	(60–76)
Pulse (beats per minute)	80	84
	(70–91)	(75–94)
Height (Meters)	1.7	1.6
	(1.6–1.8)	(1.5–1.7)
Weight (Kg)	62	59
	(57–69)	(52–66)
BMI (Kg/m^2^)	21	22
	(19–23)	(20–25)
Creatinine (µmol/L)§	74	59
	(63–86)	(51–69)
eGFR (mL/min per 1.73 m^2^)§	129	125
	(108–155)	(104–150)
Urban	2,199	3,747
	(51.2)	(47.4)
Stage 3 CKD or Worse§	34	47
	(2.3)	(1.9)
Death or lost to follow up	1,114	1,961
	(26.0)	(24.8)
**HIV-Related Characteristics**		
CD4 (count per mm^3^)	338	405
	(223–468)	(272–545)
On ART	3,091	4,960
	(72.0)	(62.8)
Use of PI???	226	295
	(5.3)	(3.7)
Length on PI of those on PIs (days)	612	609
	(261–854)	(261–861)
Time on PI>540 of those on PIs (days)	123	161
	(54.4)	(54.6)
**Cardiovascular (CV) Risk Factors**		
Prehypertension*	765	1,075
	(18%)	(14%)
Hypertension¶	481	583
	(11%)	(7%)
SBP≥140 mm Hg	392	453
	(9.1%)	(5.7%)
DBP≥90 mm Hg	262	365
	(6%)	(5%)
Overweight/Obese	453	1,782
	(11%)	(23%)
Number of CV risk factors±		
One	600	1,817
	(14%)	(23%)
Two	192	258
	(5%)	(3%)
Three	41	89
	(1%)	(1%)
At least 1 CV Risk Factor	833	2,164
	(19%)	(27%)

**BP**: blood pressure. **BMI** : body mass index. **eGFR**: estimated glomerular filtration rate. **CKD**: chronic kidney disease. **PI**: protease inhibitor. **ART:** anti-retroviral therapy.

§ The sample size was smaller for males (n = 1,471) and females (n = 2,494).

??? The sample size was smaller for males (n = 3,078) and females (n = 4,932).

*Prehypertension is defined as SBP 120–139 mmHg or DBP 80–89 mmHg.

¶ **Hypertension** is defined as SBP≥140 mmHg or DBP≥90 mmHg. Use of anti-hypertensive medications was not routinely assessed. **Overweight/Obese** is defined as BMI≥25 kg/m^2^.

± Based on the following factors: SBP≥140 mmHg, DBP≥90 mmHg and BMI≥25 kg/m^2^.

Table 2 shows the distribution of cardiovascular risk and clinical factors within CD4 count and across age categories for men. Among men, SBP was progressively higher among patients with a CD4 count >350 compared to those with a CD4 count 200–350 and <200 for the 16–35 years group (Cochrane-Armitage test for trend, p<0.05) and the 36–45 years group (p<0.05) but not in the >45 years group (p = 0.34). There were no clinically significant differences seen in DBP with higher CD4 category. There was a modest statistically significant higher BMI present in individuals with higher CD4 counts within each age category. The prevalence of hypertension tended to be higher among individuals with higher CD4 counts in the youngest age group, but not in the two older groups. The prevalence of hypertension was 6 times greater among men aged 16–35 years with CD4 greater than 200–350 compared to men with lower CD4 counts (1.4% vs. 7.8%, p = 0.02). Blood pressure was higher, in general, among men who were older.

**Table 2:** Prevalence of Cardiovascular Risk Factors Stratified by CD4 count and Age among Men, mean (SD) or %.

	CD4<200			CD4 200–350			CD4>350		
	(n = 868)			(n = 1,400)			(n = 2,025)		
	Age in years			Age in years			Age in years		
Risk Factor	16–35	36–45	>45	16–35	36–45	>45	16–35	36–45	>45
	(n = 144)	(n = 360)	(n = 364)	(n = 208)	(n = 582)	(n = 610)	(n = 383)	(n = 818)	(n = 824)
SBP, mmHg	110*	113*	117	114	115	118	114	115	118
	(12)	(15)	(17)	(15)	(15)	(18)	(13)	(13)	(17)
DBP, mmHg	67	70	70	69	70	72	69	71	72
	(9)	(10)	(11)	(9)	(10)	(11)	(10)	(10)	(11)
BMI, kg/m^2^	20.4¶	20.5¶	20.6¶	20.9	21.0	21.5	21.1	21.5	21.5
	(2.6)	(3.0)	(3.1)	(2.8)	(2.8)	(3.2)	(2.6)	(3.0)	(3.3)
Days on PI	258	417*	461	223	382	606	609	658	597
	(224)	(322)	(347)	(287)	(358)	(319)	(417)	(311)	(334)
Hypertension	1*	9	13	8	9	16	8	9	16

*p<0.05 comparing trend across each CD4 count category within an age category.

¶ p<0.01 comparing trend across each CD4 count category within an age category.

Table 3 shows the distribution of cardiovascular risk and clinical factors within CD4 count and across age categories for women. Among women, SBP was significantly higher in each successively higher CD4 count category for each age category (Table 3). There were no clinically significant differences in BMI or DBP across CD4 categories. The prevalence of hypertension was not significantly higher among women with higher CD4 counts in any of the age categories. As, expected blood pressure was higher among women who were older.

**Table 3:** Prevalence of Cardiovascular Risk Factors Stratified by CD4 count and Age among Women, mean (SD) or %.

	CD4<200			CD4 200–350			CD4>350		
	(n = 1068)			(n = 2,074)			(n = 4,759)		
	Age in years			Age in years			Age in years		
Risk Factor	16–35	36–45	>45	16–35	36–45	>45	16–35	36–45	>45
	(n = 313)	(n = 453)	(n = 302)	(n = 570)	(n = 839)	(n = 665)	(n = 1,338)	(n = 1,925)	(n = 1,496)
SBP, mmHg	108¶	109¶	113*	109	112	115	110	112	116
	(12)	(13)	(16)	(12)	(14)	(19)	(11)	(13)	(17)
DBP, mmHg	67	68¶	70	68	69	71	68	69	71
	(9)	(9)	(11)	(9)	(10)	(12)	(9)	(9)	(11)
BMI, kg/m^2^	21.4¶	21.8¶	22.2¶	22.3	22.4	22.3	22.5	22.8	22.9
	(3.4)	(3.7)	(4.2)	(3.7)	(3.8)	(3.9)	(3.7)	(3.9)	(4.2)
Days on PI	296	389¶	326¶	351	424	555	347	597	622
	(293)	(335)	(299)	(345)	(325)	(341)	(370)	(343)	(343)
Hypertension	2	3	10	4	7	15	3	6	13

* p<0.05 comparing trend across each CD4 count category within an age category.

¶ p<0.01 comparing trend across each CD4 count category within an age category.

Variables associated with greater number of cardiovascular risk factors were entered into logistic regression models for men and women separately using number of risk factors and measures of hypertension as the outcome variables (Table 4). In an age-adjusted model, CD4 count above 200 was positively associated with a greater number of cardiovascular risk factors in men (OR 1.34, 95% CI 1.10–1.63) and in women (OR 1.48, 95% CI 1.26–1.73). After including overweight/obesity into the model, the odds of having hypertension were greater than two times higher in men if they were overweight/obese (OR 2.41, 95% CI 1.88–3.09) while the relationship between CD4 count and hypertension had a smaller estimate of effect and was not statistically significant (OR 1.23, 95% CI 0.95–1.58). A similar positive relationship existed between overweight/obesity and both SBP and DBP independently. Among women, age (OR 2.21, 95% CI 1.95–2.50) and being overweight/obese (OR 1.80, 95% CI 1.50–2.16) were most strongly associated with having hypertension. Being overweight/obese almost doubled the odds that a woman would have hypertension. Age, however, was the strongest predictor of hypertension or SBP≥140 mmHg (OR 2.42, 95% CI 2.10–2.80) in the fully adjusted model.

**Table 4:** Factors associated with higher levels of cardiovascular risk.

Men (n = 4293)				
	Number of Risk Factors	HTN*	SBP≥140 mmHg	DBP≥90 mmHg
Age	1.58 (1.41–1.76)	1.62 (1.40–1.87)	1.77 (1.50–2.08)	1.50 (1.24–1.81)
CD4 Count ≥200	1.34 (1.10–1.63)	1.23 (0.95–1.58)	1.12 (0.86–1.48)	1.19 (0.86–1.66)
BMI≥25 kg/m^2^	-	2.41 (1.88–3.09)	2.35 (1.79–3.07)	2.50 (1.83–3.41)

Women (n = 7901)				
	Number of Risk Factors	HTN	SBP≥140 mmHg	DBP≥90 mmHg
Age	1.38 (1.26–1.48)	2.21 (1.95–2.50)	2.42 (2.10–2.80)	2.04 (1.76–2.39)
CD4 Count ≥200	1.48 (1.26–1.73)	1.54 (1.15–2.07)	1.50 (1.08–2.09)	1.60(1.10–2.33)
BMI≥25 kg/m^2^	-	1.80 (1.50–2.16)	1.72 (1.40–2.11)	2.0 (1.60–2.50)

Age is a categorical variable defined by three age categories: 16–35, 36–45 and <45 years.

CD4 count is a binary variable: ≥200 and <200 cells/mm^3^.

BMI was not included as a covariate in the risk factor analysis because BMI≥25 kg/m^2^ is one of the cardiovascular risk factors.

* HTN: hypertension.

In subsequent models, we restricted our analyses to only patients who were prescribed PIs (Table 5). Length of time on PIs≥540 days was not found to be significantly associated with higher SBP, DBP, hypertension or greater number of cardiovascular risk factors. Of the four variables included in the logistic regression models using blood pressure as the outcome, overweight/obesity was the strongest predictor of hypertension and higher SBP.

**Table 5:** Factors associated with higher levels of cardiovascular risk among patients using protease inhibitors.

Men (n = 226)				
	Number of Risk Factors	HTN*	SBP≥140 mmHg	DBP≥90 mmHg
Age	1.51 (0.88–2.60)	1.80 (0.81–3.98)	1.99 (0.81–4.91)	1.99(0.69–5.79)
CD4 Count ≥200	2.47 (1.21–5.07)	1.17 (0.42–3.26)	1.35 (0.43–4.24)	0.62(0.16–2.35)
Days on PI≥540	1.66 (0.80–3.43)	1.82 (0.63–5.27)	1.62 (0.50–5.20)	1.62(0.42–6.20)
BMI≥25 kg/m^2^	-	4.92 (1.83–13.21)	5.43 (1.89–15.58)	5.91 (1.67–20.89)

Women (n = 295)				
	Number of Risk Factors	HTN	SBP≥140 mmHg	DBP≥90 mmHg
Age	1.61 (1.10–2.38)	2.84 (1.35–5.96)	2.64 (1.21–5.78)	2.45 (0.98–6.11)
CD4 Count ≥200	2.10 (1.11–3.97)	1.32 (0.44–3.99)	1.05 (0.34–3.25)	5.57(0.69–45.18)
Days on PI≥540	1.09 (0.62–1.90)	1.18 (0.47–2.98)	1.13 (0.42–3.04)	1.17 (0.37–2.65)
BMI≥25 Kg/m^2^	-	3.32 (1.39–7.94)	3.26 (1.28–8.33)	2.28 (0.79–6.58)

Age is a categorical variable defined by three age categories: 16–35, 36–45 and <45 years.

CD4 count is a binary variable: ≥200 and <200 cells/mm^3^.

BMI was not included as a covariate in the risk factor analysis because BMI≥25 kg/m^2^ is one of the cardiovascular risk factors.

* HTN: hypertension.

## Discussion

Cardiovascular diseases are a widely recognized complication of HIV infection [23]. Very few studies of the distribution of cardiovascular risk factors among HIV+ individuals have examined sub-Saharan African populations. We have found an 11.2% prevalence of hypertension among Kenyan HIV+ men and 7.4% prevalence among Kenyan HIV+ women. Overweight/obesity was also prevalent among men (10.6%) but higher among women (22.6%). These data, to our knowledge, represent the first report of CVD risk factors from an HIV cohort of this size in sub-Saharan Africa.

### Hypertension and HIV

With respect to hypertension among HIV+ individuals, little is known of the epidemiology in sub-Saharan Africa. Developed country studies suggest the prevalence of hypertension in HIV+ individuals is between 8 and 39% [24], [25]. Our prevalence estimates may be lower than reported in developed countries due to the low population rate of hypertension in the region. The most recent population based hypertension prevalence data for Kenya are from 1987 and suggest a rate between 1.2% and 2.2% [26]. If the population estimates reflect the true prevalence in the population, our findings would suggest a higher than expected rate of hypertension among Kenyan HIV+ individuals. This is consistent with observations that HIV can have effects on the vasculature of infected individuals at a relatively young age [27]. To put our findings in context would require a contemporary analysis of the prevalence of hypertension in Western Kenya.

The relationship between immune system function and hypertension in HIV+ individuals continues to be the subject of ongoing investigation [28]. Two large studies have suggested an association between a lower CD4 count and incident cardiovascular events [11], [29]. This relationship is posited to be mediated by a chronic pro-inflammatory state which promotes atherosclerosis [30]. However, individual cardiovascular risk is usually determined by a combination of several risk factors including age, family history, smoking, hypertension, overweight, diabetes and dyslipidemia. With regard to blood pressure alone, better immune system function as measured by a higher CD4 count is related to higher blood pressure [31]. The pathophysiology in this case has not been well established but is thought to be due to improved overall general health and nutritional status [32]. Obesity has also been implicated as a potential confounder [32], [33]. In general, however, the literature on the relationship between immune function and blood pressure in HIV+ individuals has mostly emphasized the impact of class of ART [34], [35], [36]. In the present analysis, the relationship between higher CD4 count and higher prevalence of hypertension was most dramatic among young men while the relationship between CD4 count and hypertension seemed blunted among the older age groups. One possible explanation is that the specific effects of HIV on the vascular system are most noticeable in patients with few other traditional cardiovascular risk factors. Indeed, hypertension is more common among young people with HIV [25]. As age increases, the attributable effects of HIV on the vascular system may be smaller than the effects of age or other factors.

### Overweight/Obesity and HIV

We have found that one of every ten HIV+ Kenyan men in our sample was overweight/obese and that overweight/obesity was a stronger predictor of hypertension than age. Findings from developed countries suggest that PI use may be driving both hypertension and obesity [37]. However, in contrast to studies from developed countries, it is unlikely that use of protease inhibitors were a major cause of obesity in our sample since the proportion of people using PIs was low (6.5%). Moreover, our logistic regression analyses failed to show a precise relationship between length of time taking PIs and hypertension. Instead, metabolic disturbances related to HIV infection, other ART regimens, diet or other factors may be responsible.

The prevalence of overweight/obesity among women in this analysis is similar to that of women in the general Kenyan population [38]. However, it is not clear that, in the general population of black women, overweight/obesity has a consistent deleterious effect. Historically, black women do not have the same risk of death associated with obesity as women of other ethnic groups [39] despite higher rates of diabetes and hypertension among black women [40]. It has also been suggested that overweight/obesity is beneficial and is associated with a lower risk of death among HIV+ women [41]. In the modern era, due to an epidemiologic transition in sub-Saharan Africa, it is reasonable to expect that the type and consequences of fat distribution seen in African HIV+ women may be different from those previously reported [42]. While overweight/obesity among HIV+ women is not strongly related to hypertension in our cross-sectional analysis, the long-term clinical effects of overweight/obesity in HIV should be studied with attention to sex-based differences.

### Protease inhibitors and HTN

Protease inhibitor therapy is known to have cardiovascular consequences and CVD risk is greatest with prolonged (≥540 days) exposure [11], [12]. However, in our multivariate regression analyses adjusted for age and CD4 count, the estimated association between a longer duration on PIs and higher cardiovascular risk included the null. This finding does not support an independent relationship between PI use and higher cardiovascular risk. Regarding the relationship between PI therapy and elevated blood pressure, we also do not find a precise statistically significant relationship. Use of PIs is related to greater risk of myocardial infarction in large studies [12], [13] and our lack of an association between PI use and number of CVD risk factors may have been due to small sample size or the lack of data on cardiovascular events. Among patients on PIs, we did find a significant relationship between overweight/obesity and higher blood pressure as there is in the general population and in HIV+ patients in developed countries [33] suggesting that the relationship between hypertension and weight is also preserved among those on PIs. Longitudinal data on cardiovascular events among patients on PIs in Kenya, would be necessary to identify whether a relationship exists between PI use and CVD in this region and to clarify the effect of overweight/obesity or other factors on CVD in this subgroup.

There are several limitations within this analysis that deserve mention. This was a retrospective analysis of clinical data and the variables of interest were not routinely measured in standard, protocol-driven manner. Our analysis is therefore limited by selection bias potentially introduced at the time of the clinical encounter in that only patients who were able to come to clinic visits were included in this analysis. As a result we may have overestimated the prevalence of hypertension in this population because blood pressure is more likely to have been measured in patients suspected to have high blood pressure. A large number of participants were excluded from the analysis due to having too few measures of blood pressure during the follow-up period which limits the external validity of our findings and is related to the retrospective nature of the study design. Another limitation of this analysis is that the data was based on measurements taken at one point in time according to clinical indications and were assumed to reflect their chronic condition. Moreover, our analysis of cardiovascular risk factors is limited to hypertension and overweight/obesity. Other cardiovascular risk factors such as tobacco use, diabetes, physical activity and dietary factors were not routinely collected during the clinical encounters during this time period. In addition, measurement of blood cholesterol was usually performed as clinically indicated and only on a small portion of patients. Hypertension, however, is the most important risk factor for myocardial infarction among Africans [43] and actionable information on the prevalence of hypertension among HIV+ individuals in Africa has been lacking. This information is critical if we are to expand health care systems in sub-Saharan Africa and create stronger interactions with existing major global HIV initiatives. A recent Institute of Medicine report calls for such approaches that dovetail with current efforts to transition from costly, disease-specific approaches toward more efficient approaches that promote better primary health care to meet a range of health needs [44].

In conclusion, much of the literature surrounding cardiovascular disease among HIV+ patients in sub-Saharan Africa has focused on infectious and inflammatory manifestations of HIV-associated cardiac disease. There are, however, good reasons to expect a transition in the magnitude of degenerative cardiovascular disease among HIV+ patients in sub-Saharan Africa. These reasons include the changing patterns of traditional cardiovascular risk factors in the region, ageing of the population, urbanization, and increasing prolonged exposure to ART. This analysis has shown that hypertension and obesity are already highly prevalent among HIV+ patients in Western Kenya. The public policy implications are great. There is a mismatch between aggregate spending for global health and overall disease burden, whereby the least amount of spending goes towards non-communicable diseases despite these diseases contributing to the largest number of disability adjusted life-years and overall mortality [45]. One approach to address this discordance is by expanding the scope of HIV care programs to recognize the important overlap between communicable and noncommunicable diseases [46]. Programs in sub-Saharan Africa that focus solely on HIV care are missing a major opportunity to improve population health status at a substantial future cost.
